# Sex hormones affect the pathogenesis and clinical characteristics of systemic lupus erythematosus

**DOI:** 10.3389/fmed.2022.906475

**Published:** 2022-08-11

**Authors:** Ji-Won Kim, Hyoun-Ah Kim, Chang-Hee Suh, Ju-Yang Jung

**Affiliations:** ^1^Department of Rheumatology, Ajou University School of Medicine, Suwon, South Korea; ^2^Department of Molecular Science and Technology, Ajou University, Suwon, South Korea

**Keywords:** systemic lupus erythematosus, sex hormone, clinical characteristic, pathogenesis, hormone therapy

## Abstract

Systemic lupus erythematosus (SLE) affects women more frequently than men, similar to the female predilection for other autoimmune diseases. Moreover, male patients with SLE exhibit different clinical features than female patients. Sex-associated differences in SLE required special considerations for disease management such as during pregnancy or hormone replacement therapy (HRT). Sex hormones, namely, estrogen and testosterone, are known to affect immune responses and autoimmunity. While estrogen and progesterone promote type I immune response, and testosterone enhances T-helper 1 response. Sex hormones also influence Toll-like receptor pathways, and estrogen receptor signaling is involved in the activation and tolerance of immune cells. Further, the clinical features of SLE vary according to hormonal changes in female patients. Alterations in sex hormones during pregnancy can alter the disease activity of SLE, which is associated with pregnancy outcomes. Additionally, HRT may change SLE status. Sex hormones affect the pathogenesis, clinical features, and management of SLE; thus, understanding the occurrence and exacerbation of disease caused by sex hormones is necessary to improve its management.

## Introduction

Systemic lupus erythematosus (SLE), a chronic autoimmune inflammatory disease with heterogeneous clinical manifestations and course, affects multiple tissue and organ systems with, varying severity depending on the patient and duration of illness (1). Although many aspects of its etiology remain unclear, SLE is a complex disease known to result from the aberrant activity of the immune system due to environmental, genetic, epigenetic, and hormonal factors (2). Like other autoimmune diseases, such as rheumatoid arthritis, Sjogren’s syndrome, and systemic sclerosis, SLE has a much higher prevalence in women than in men, with a female-to-male ratio ranging from 8:1 to 15:1 (3, 4). The striking difference in prevalence appears during the peak reproductive age, whereas female predominance significantly decreases during pre-puberty and post-menopause (5).

Factors associated with sex differences in SLE include sex chromosome genes, sex-dependent environmental factors, and gut microbiome composition, but considerable evidence supports that sex hormones are a major factor (6). As previously mentioned, SLE is typically more prevalent in young women of childbearing age. Indeed, its prevalence in women is only double that in men during childhood and postmenopausal periods (7). Furthermore, the disease activity of SLE can vary depending on hormonal changes such as the menstrual cycle and menopause, with a flare rate of 45–70% in pregnancy (8). In some patients with SLE, symptoms worsen each month as menstruation begins, and estrogen-containing therapies, namely, oral contraceptives (O) and postmenopausal hormone replacement therapy (HRT), are associated with an increased risk of flare (9, 10). In addition, the frequency and severity of flares decrease in most menopausal patients with SLE (11). In a lupus-prone model in NZB × NZW F1 mice, disease development was prevalent and survival time was shorter in women than in men (12).

Sex hormones include estrogen, progesterone, testosterone, dihydrotestosterone, and dehydroepiandrosterone (13). Estrogen, progesterone, and androgens are produced by the ovary in women, while testosterone precursors are produced mostly by Leydig cells in the testis in men and in the adrenal gland and thecal cells of the ovary in women (14). In women, estrogen and progesterone levels fluctuated during the menstrual cycle and life span, namely, premenopause and menopause, whereas testosterone levels remain steady during the menstrual cycle and decrease after menopause. Serum testosterone levels are higher in men than in women and decrease with age. Estrogen mainly affects reproductive function and additional processes, such as bone mass and fat distribution, while testosterone regulates physiological processes in muscle mass and strength, bone mass, fat distribution, and production of sperm and red blood cells.

In addition, sex hormones are involved in the development and function of innate and adaptive immune responses, and dysregulation of these mechanisms contributes to autoimmune abnormalities (15). Progesterone and androgens mainly have immunosuppressive and anti-inflammatory effects, thus protecting against autoimmune diseases, whereas estrogen is generally regarded as pathogenic due to its immune-stimulatory effects (16). Considering the mechanisms underlying altered immune responses and female predilection, estrogen is widely perceived as contributing to the predisposition for SLE. Recently, various clinical studies have reported sex-dependent genetic and epigenetic changes in SLE, revealing the complex role of sex hormones in addition to estrogen in the pathogenesis of SLE (17–19).

Herein, we review the current evidence regarding the role of sex hormones in the pathogenesis of SLE and describe the clinical features according to sex hormonal changes.

## Methods

A systemic search of all English-language studies was performed in the Medline/PubMed, Scopus, and EMBASE databases using the following keywords: “sex hormone,” “sex steroid,” “systemic lupus erythematosus,” “autoimmune disease,” “estrogen,” “progesterone,” “testosterone,” and “sex differences,” as well as their abbreviations. Additionally, all related studies were searched manually for relevant keywords such as “oral contraceptives” and “hormone replacement therapy.” Abstracts from relevant studies were reviewed, and appropriate articles were retrieved, and non-relevant papers and duplicate studies were excluded. All authors of this study conducted searches and articles were reviewed independently.

## Results and discussion

### Influence of sex hormones on immune response or autoimmunity (Figure 1)

#### Estrogen

Immune cells express two estrogen receptor (ER) subtypes, ERα and ERβ, and activation of ER-mediated or ER-independent pathways controls immune responses. ER subtypes and their mechanisms of action vary depending on the cell or environment, while hormone’ concentration, density, distribution, and receptor, subtype affect immune responses. Gene expression analysis demonstrated that the expression of ERα mRNA was increased, while that of ERβ was decreased in peripheral blood mononuclear cells (PBMCs) from patients with SLE compared to healthy controls (20). However, no unique variants in ERα, ERα splice variants, and ERβ were identified in PBMCs from 19 patients with SLE compared to 12 healthy individuals (21). Furthermore, the depletion of ERα attenuated the development of glomerulonephritis and anti-double-stranded DNA (dsDNA) antibodies, while prolonging the survival of NZB × NZW F1 mice, whereas ERβ deficiency had no effect on lupus manifestations (22, 23). Currently, the association between ER expression/action and SLE remains unclear.

**FIGURE 1:**
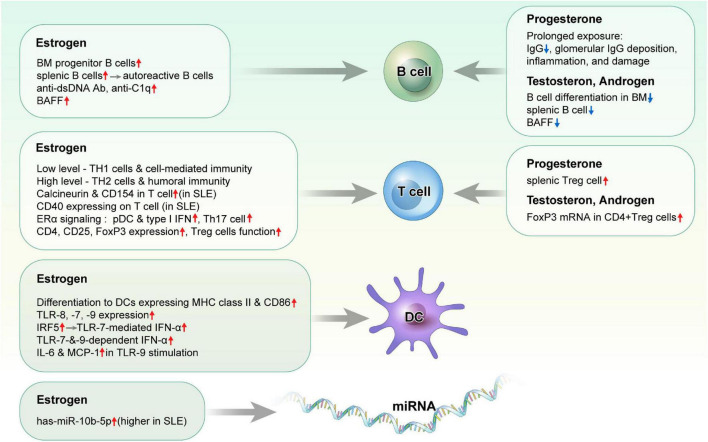
Effects of sex hormones on immune cells in systemic lupus erythematosus.

##### B cells and autoantibody production

Estrogen stimulates B cell maturation and antibody secretion in the normal immune system. With respect to its impact on the autoimmune response, estrogen has been shown to increase the abundance of bone marrow progenitor B cells and enhance the survival of splenic B cells, which promotes the development of autoreactive B cells (24). In one study, estradiol (E2) treatment increased the production of anti-dsDNA antibodies and IgG in PBMCs from patients with active SLE, but not in patients with inactive disease or in a normal population (25). Additionally, E2 administration induced a lupus phenotype in BALB/c mice that expressed a transgene-encoded H chain of an anti-DNA antibody, rescued high-affinity DNA-reactive B cells, and led to increased Bcl-2 expression, which improved the survival of autoreactive B cells (26, 27). Meanwhile, tamoxifen treatment reduced levels of autoantibodies to IgG3, prolonged survival time, and was associated with weaker glomerular immune complex deposition in NZB × NZW F1 mice (28). Further, treatment with E2 increased levels of B cell-activating factor (BAFF) in immune cells, namely, macrophages, which promoted the survival of autoreactive B cells and autoantibody production, while ERα-knockout (KO) splenic cells showed decreased BAFF expression (29). In addition, NZB × NZW F1 mice treated with E2 exhibited more severe proteinuria and histological change in the glomerular tissue, along with increased levels of anti-C1q and anti-dsDNA antibodies (30).

##### T cells

Low estrogen levels enhance T-helper type 1 (TH1) cells and cell-mediated immunity, whereas high estrogen levels promote T-helper type 2 (TH2) cells and humoral immunity. E2 treatment was shown to enhance the expression of calcineurin, a T cell activation marker that acts through ERs, in the T cells of female patients with SLE, but not in those of HCs or male patients with SLE. Consistently, treatment with ERα and ERβ agonists increased the expression of calcineurin and CD154 in the T cells of patients with SLE (31, 32). E2 treatment stimulated T cells to express CD40 ligands in patients with SLE but not in normal women (33). E2 treatment induced the lupus phenotype, namely, nephritis, and production of TH2 cytokines and autoantibodies in wild-type mice, but not or minimally in ERα-KO mice (34). CD4-ERα KO mice had increased autoantibody production and CD4 + CD44 + CXCR5 + Bcl-6 + follicular helper T (TFH) cells, and E2 treatment decreased TFH cell responses, antigen-specific antibody production, and reduced IL-21 and Bcl-6 expression (35).

While different results had been reported in the populations of regulatory T (Treg) cells in SLE, incubation with E2 resulted in increased CD4, CD25, and FoxP3 mRNA expression in PBMCs from a healthy female, those from patients with SLE exhibited reduced FoxP3 mRNA expression (36). The deficiency of estrogen-related receptor γ (Esrrg), a murine lupus susceptibility gene associated with CD4 + T cell activation, has been shown to result in impaired function of Treg cells (37, 38). In addition, levels of human ESRRG, which is highly expressed in Treg cells, were reportedly lower in CD4 + T cells of patients with SLE than in those of HCs.

While increased population of CD4 + Th17 cells and IL-17A production has a pathogenic role in SLE, E2 treatment showed an inhibitory effect in Th17 cell differentiation in CNS autoimmunity (39). In addition, ERα KO mice showed TH1 and Th17 cell differentiation with IL-17 production, and estradiol inhibited Th17 cell differentiation through the downregulation of RORγt transcription (40). IL-17A production and IL-23R expression were increased in Th17 cells from female mice compared to those from male mice, both of which were decreased and Th17 cell proliferation was downregulated when ERα expression was suppressed (41).

##### Dendritic cells and toll-like receptor pathways

Dendritic cells (DCs), especially plasmacytoid DCs (pDCs), are activated in SLE to produce type I IFN through the Toll-like receptor (TLR)-7 or TLR-9 pathway through which endogenous DNA and RNA provoke autoimmune responses as self-antigen. Several DC subsets, namely, pDCs, express different patterns of ERs and affect ERα signaling (42). E2 treatment promoted the differentiation of DCs expressing high levels of cell surface MHC class II and CD86, whereas ER antagonists blocked DC differentiation (43). ERα deficient lupus-prone mice (strain NZM2410) had decreased frequency of pDCs and reduced endogenous expression of MHC-II and PDC-TREM, which modulates type I IFN production (44).

TLR-7-mediated IFN-α production was reportedly increased in the peripheral blood lymphocytes of healthy females compared to those of healthy males (45). E2 therapy enhanced TLR-7- and TLR-9-dependent production of IFN-α stimulated by nucleic acid-containing complexes in pDCs from postmenopausal women, while TLR-medicated IFN-α production by pDCs was restored in ERα-KO mice by E2 treatment (46). In another study, TLR-9 induced IL-6 and MCP-1 production by DCs was decreased in ERα-KO lupus-prone mice (strain NZM2410), and IL-1β and IL-23 expression were induced by a TLR-9 agonist in wild-type but not ERα-KO mice (47). Moreover, the delivery of recombinant IFN regulatory factor 5 (IRF5) protein into human pDCs increased TLR-7-mediated IFN-α secretion, while the genetic ablation of the estrogen receptor 1 gene in pDCs reduced irf5 mRNA expression as well as IFN-α production (48). Further, estrogen treatment enhanced the expressions of TLR-8 and endosomal TLR-7 and TLR-9 in the PBMCs of patients with SLE compared to that in the PBMCs of HCs (49). Additionally, E2 exposure exacerbated proteinuria and glomerular immune complex deposition in female lupus-prone MRL^lpr^ mice through the induction of TLR-7 and -9 expression on splenic leukocytes and CD19 cells (50). Estrogen treatment was shown to enhance the expression of STAT1, which induces IFN-stimulated gene expression and upregulates TLR-8 expression (51). These data suggest that estrogen influences DC activation and IFN production through the TLR signaling pathways.

##### Epigenetic modulation

There were some reports finding the epigenetic changes related to the effects of estrogen or ER expression in SLE. DNA demethylation within the proximal promotor region relative to the transcription star site of the human ERα gene was associated with the overexpression of the ERα gene in SLE (52). And E2 inhibited DNA methyltransferase 1 (DNMT1) and enhances global DNA hypomethylation in SLE CD4 + T cells (53). ER agonists rescued downregulated DNMT1 and DNA hypomethylation.

E2 treatment enhances the activation of IFN-α signaling in SLE B cells *via* inhibitor of kappa B kinase ε (IKKε) by downregulating the expressions of let-7e-5p, miR-98-5p, and miR-145a-5p (54).

Estrogen treatment induced the overexpression of has-miR-10b-5p in T cells, and has-miR-10b-5p suppresses serine/arginine-rich splicing factor 1, which controls genes involved in T cell signaling and cytokine production. Has-miR-10b-5p expression was elevated in T cells from healthy women than healthy men, and elevated in T cells from patients with SLE, regardless of sex and SLE disease activity index (SLEDAI) (55).

#### Progesterone

Progesterone is a female reproductive steroid with immunomodulatory functions. Prolonged exposure to medroxyprogesterone acetate, synthetic progesterone used for contraception, led to lower serum IgG level, and decreased mortality in female NZB × NZW mice, although such treatment did not affect lupus phenotypes in other studies (56–58). The action of progesterone depends on its receptors, namely, progesterone receptor (PR), glucocorticoid receptor, and membrane PR. While low progesterone levels activate PRs and membrane PRs, high levels can bind not only to PRs and membrane PRs but also to glucocorticoid receptor, which is critical for reproduction. In one study, aged female PR-KO lupus-prone Nba2 mice exhibited increased IgG autoantibody production, and glomerular IgG deposition, inflammation, and damage compared to male mice (59). In addition, knockout of PR resulted in a lower splenic Treg cell population, but an increased proportion of follicular Th cells in aged female Nba2 mice.

#### Testosterone

Testosterone inhibits B cell differentiation in the bone marrow. Knockout of male androgen receptor, a testosterone receptor, resulted in increased levels of bone marrow B cell precursors in mice (60). In addition, male mice lacking androgen receptors had higher splenic B cell population and serum BAFF levels (61). Further, levels of plasma androgens, namely, testosterone and androstenedione, were lower in female patients with active SLE (62).

While some studies have reported the therapeutic effect of testosterone and other androgens against SLE disease activity, testosterone patches and 19-nortestosterone failed to improve disease activity or quality of life in patients with SLE (63–66). *In vitro* study assessing the effect of sex hormones on changes in Treg cells demonstrated that androgen/testosterone enhanced FoxP3 mRNA expression in CD4 + Treg cells of patients with SLE (36).

### Influence of menstrual cycle on systemic lupus erythematosus

During the menstrual cycle, follicle-stimulating hormone (FSH) stimulates the ovarian follicles to produce E2. Thus, the plasma E2 concentration is increased, while that of progesterone is decreased in the follicular phase, and both E2 and progesterone plasma concentrations are high in the luteal phase (36). Increased E2 levels lead to a mid-cycle surge of luteinizing hormone, which initiates ovulation; if fertilization does not occur, progesterone levels drop. Luteolysis is inhibited during pregnancy, leading to prolonged high E2 and progesterone levels. In addition, E2 and progesterone levels are low during menopause along with the depletion of follicles.

A study comparing reproductive health histories between patients with SLE, and the general population reported no differences in hormone levels throughout the menstrual cycle (67). However, menopause occurred earlier in patients with SLE, and the use of HRT was frequent, and the family size was reduced in patients with lupus nephritis. Some data have shown that premature ovarian dysfunction is more common in patients with SLE than in HCs and is associated with the use of cyclophosphamide (68, 69). Compared to 30 HCs, abnormal and longer-length menstrual cycles were more frequent, and the median FSH level was higher, and that of progesterone was lower in 30 patients with juvenile SLE (70). A study analyzing a self-reported survey of patients with SLE revealed higher pain, fatigue, and disease activity during menses than during the hormonal surge phase, although recall bias and confusion with pre-menstrual syndrome existed (71). In examining the influence of the menstrual cycle or fertility on SLE, disease activity, use of medication (including glucocorticoids and cyclophosphamide), and individual differences should be considered.

Analysis of PBMCs has revealed significant changes in gene expressions, including that of TNF superfamily member 14 and signal regulatory protein-γ, during the menstrual cycle of patients with SLE compared to that of HCs (72). A study analyzing the expression of sex hormone receptor genes in PBMCs, and cell subsets reported that several immune response genes were more highly expressed during the ovulatory and mid-luteal phase (73). In addition, the level of sex hormone-binding globulin, a steroid hormone transport protein, was correlated with ERβ1 gene expression.

### Characteristics of male systemic lupus erythematosus

#### Sex hormones and chromosomes in male systemic lupus erythematosus

Unsurprisingly, the role that sex hormones play in male and female SLE differs significantly, which has been demonstrated both in murine models and clinical studies (74). In the NZB × NZW F1 mouse model, an autoimmune disease resembling human SLE is characterized by high levels of antinuclear and anti-dsDNA antibodies, lymphadenopathy, splenomegaly, and immune complex-mediated glomerulonephritis. NZB × NZW F1 mice administered androgens exhibited reduced lupus-like phenotypes and improved survival rates, whereas autoantibodies and accelerated autoimmune disease appeared earlier in castrated NZB × NZW F1 mice administered estrogen (75, 76). A recent study with a lupus-prone mouse model reported that a male-driven immunoinhibitory milieu was related to B cell activation and differentiation, and ultimately delayed or prevented lupus-like disease, suggesting that androgens affect lupus pathogenesis and exert different therapeutic effects in males and females (77). Given the clear inhibitory effects of androgen on the immune system, a recent interest is to discover SLE therapies targeting immunomodulatory cells by elucidating mechanisms that affect the number and functionality of these cells such as regulator T/B cells, MDSCs, and M2 macrophages in genetically predisposed male mice (78).

In human studies, significantly lower androgen levels, which were inversely related to hypoandrogenism, testosterone levels, and disease activity, were detected in male patients with SLE compared to HCs (79–81). As in murine models, the clinical symptoms and serological features of men and women respond differently to synthetic androgen therapy as a treatment for SLE. Two studies have reported that testosterone supplementation improved the clinical symptoms of male lupus patients with Klinefelter’s syndrome (82, 83). Additionally, testosterone injections for cross-gender therapy resolved the skin symptoms of a transgender patient (female to male) with subcutaneous lupus erythematosus (84). In contrast, a small clinical trial reported that men treated with 19-nortestosterone exhibited decreased testosterone levels, increased serum anti-dsDNA antibody levels, and new clinical features, namely, Raynaud’s phenomenon and pleuropericardial disease (64). In another clinical trial, testosterone patches did not significantly affect disease activity, quality of life, or sexual function in male patients with SLE (63). The clinical efficacy of androgen treatment in male patients with SLE remains unclear, and further studies are warranted to determine whether such treatments should be more widely provided.

In addition to sex hormones, there is growing evidence that the role of the X chromosome helps explain whether more women than men develop autoimmune diseases, namely, SLE (85–87). The first evidence supporting that the factors associated with X chromosomes cause susceptibility for SLE was a report that the prevalence of Klinefelter’s syndrome (karyotype 47, XXY) is increased 14-fold in men with SLE compared to that in an unselected male population (85). With similar results, in one study, karyotype 47, XXX in females predicted an approximately 25-fold relative risk for SLE, and another report showed that 46, XX males (de la Chapelle’s syndrome) were excessively present among males with SLE (86, 87). These data support that the number of X chromosomes, not phenotypes, is related to the cause of sex bias in SLE. Recently, genes in the X chromosome are also observed to be attributed to the female bias in SLE. The X chromosome encodes a greater number of genes than the Y chromosome, and X-linked genes such as KDM6a, TLR-7, CXorf21, and IRAK1 are found to be overexpressed in females’ autoimmunity. Furthermore, recent data suggest that X-linked genetic factors are involved in epigenetic mechanisms to avoid X chromosome inactivation, thereby enhancing female susceptibility to autoimmune diseases (88).

#### Clinical features and outcomes in male systemic lupus erythematosus

Due to the perception that SLE is a disease in women of childbearing age, appropriate diagnosis and treatment are often delayed in men. Overcoming this challenge requires an awareness of the distinctive presentation of male SLE. Studies worldwide have confirmed that the peak ages of SLE incidence and prevalence are delayed for men compared to women (89). Although considerable variability is reported according to region and race, the mean age at diagnosis is 26−55 years for men, compared to 26.3−42.6 years for women (90). The peak incidence of SLE usually occurs for women in their 20s–50s, while that for men occurs in their 50s–70s (89). The prevalence curve by age tends to be similar to the distribution of incidence data, with the peak age of prevalence ranging from 45 to 69 years for women and 40 to 89 years for men (89, 91).

Serologically, anti-dsDNA and anti-Smith antibodies have been observed to occur more frequently in male SLE, whereas some studies have reported lower levels of anti-Ro/SSA and anti-La/SSB antibodies in male SLE (92–95). Although differences occur depending on the reported group, anti-U1-RNP and anti-cardiolipin antibodies and low complement appear to occur at almost the same rates in men and women (92, 96). Lupus anticoagulant positivity is more common in men, combined with smoking and alcohol use, which is related to a higher prevalence of thrombosis in male SLE than in female SLE (96–98). Additionally, renal, hematological, and neurological involvement, as well as serositis features prominently in male SLE, leading to rapid organ damage compared to female SLE (94, 99). The increase in autoantibody production and the development of lupus nephritis in male SLE is presumed to be due to the global deletion of ERα or especially in B cells (98).

Regarding skin involvement, discoid, and subacute lesions occurred more frequently in male SLE, while malar rash, photosensitivity, and Raynaud’s phenomenon were much less common (74, 90, 92, 94–104). Musculoskeletal involvement and alopecia were reported less frequently in male SLE, with fewer outpatient visits and emergency department visits than in female SLE (93, 101, 105).

In relation to disease activity, SLEDAI scores and lupus severity of disease index scores did not differ significantly between the sexes in most studies (90, 94, 95, 98, 104). Conversely, renal damage is a major concern in male SLE, as several studies reported that male sex is a strong predictor of baseline damage and men have a high risk of developing chronic renal failure (91, 92, 105–109). In cohorts in the United States and Taiwan, male sex was associated with a 2-fold greater risk of end-stage renal disease (99, 110). However, in a recent study using the national data system in the United States, similar rates for both sexes were reported for end-stage renal disease and mortality (105). Increased incidence of cardiovascular events due to ischemic heart disease or stroke was also reported among male patients with SLE (90, 98, 100). In this regard, male SLE is considered to have a poorer prognosis than female SLE due to renal involvement and concomitant cardiovascular diseases. Although survival rates vary, most studies did not report statistically significant differences between men and women (90, 92, 95, 105).

The clinical characteristics and outcomes of SLE display notable sex differences, which are most influenced by sex hormones. However, considering age, race, national health system, and small cohorts of male patients with SLE, further studies are needed to unravel the mystery of male SLE and potential therapeutic interventions for the disease.

### Influence of hormone therapy on systemic lupus erythematosus

#### Oral contraceptives and systemic lupus erythematosus

17α-ethinyl estradiol, a synthetic analog of 17β-estradiol (E2), is a major component in OCPs and has been commonly used in hormone therapy. As the timing of pregnancy greatly contributes to success in SLE and flares cause adverse pregnancy outcomes, contraception is often considered for women with SLE, leading to the use of OCPs (111). Two major categories of OCPs, combined oral contraceptives (COCs) that contain estrogen and progesterone and progestin-only formulations, are commonly used as reversible contraceptives and are mostly safe (112). However, reflecting on the unpredictable and variable nature of SLE, OCPs have been considered unsafe and not often prescribed for women with SLE (113, 114). Several studies have reported controversial results regarding the use of OCPs in patients with SLE (Table 1). The role of exogenous estrogen as a flare factor was first reported in a case report in the 1960s (115). Early case reports and retrospective studies supported that the patients with established disease exposed to OCPs were at risk for developing SLE (105–120). A frequently cited and representative retrospective study reported by Jungers et al. found that flares occurred in 43% of patients with lupus nephritis when taking COCs, which was not observed with progestin-only formulations (117). In another retrospective study based on self-reported flares, 13% of patients experienced flares after initiating OCPs, particularly with musculoskeletal symptoms (120). Moreover, several cases of pulmonary hypertension and venous thromboembolism have been reported in patients with SLE after the use of OCPs (118, 121).

**TABLE 1:** List of studies on the risk of disease onset or flares in patients with systemic lupus erythematosus using oral contraceptives.

Study design	Study population	Oral contraceptives, dose	Main findings	References
**SLE Flares**				
Case report	23-year-old female	COC, 3 mg norethisterone + 50 μg ethinyl estradiol	Flare (high fever, arthritis, malar rash) in 1 week	(115)
Case report	Two cases	POC, Mestranol 100 μg POC, Mestranol 80 μg	Flare (arthritis) in 10 days Flare (skin rash) in 3 months	(116)
Retrospective study	26 Lupus nephritis	COC, 50 μg (14 patients) and 30 μg (7 patients) ethinyl estradiol POC (11 patients)	- Incidence of flare: 43% in COC groups within 3 months - No flare in POC group	(117)
Case report	16-year-old female	30 μg ethinyl estradiol + 150 μg levonorgestrel	Pulmonary hypertension in 7 months later	(118)
Retrospective study	85 SLE	COC (31 patients), 30 μg ethinyl estradiol + 150 μg levonorgestrel/75 μg gestodene POC (32 patients) Other unspecified	- Incidence of flare: 4 (13%) during the first 6 months - Incidence of flare was similar as in patients not using OCPs	(119)
Retrospective questionnaire study	55 SLE	OCP unspecified	Incidence of flare: 7 (13%) reported an exacerbation of disease activity, mostly musculoskeletal system	(120)
RCT, single blind, non-placebo, follow-up 12 months	162 SLE (≤ 40 years old, with mild or stable disease)	COC, 35 μg ethinyl estradiol + 150 μg levonorgestrel POC, 30 μg levonorgestrel IUD (TCu 380A copper device)	No difference among groups in mean activity, incidence of flares or time to first flare	(131)
RCT, double blind placebo-controlled, follow-up 12 months	183 stable or inactive SLE (91 OCP vs. 92 placebo)	Triphasic ethinyl estradiol 35μg + norethisterone at a dose of 0.5−1 mg for 12 cycles of 28 days	No differences between groups in occurrence of flares of any type (Severe lupus flare occurred in 7.7% of OCP group vs. 7.6% in the placebo group)	(132)
**SLE onset**				
Case report	False positive serological test for syphilis	COC, 1 mg norethisterone + 50 μg ethinyl estradiol	Developed SLE 3 weeks after the start of OCP	(122)
Case report	False positive serologic prenuptial syphilis test	1 mg ethynodiol diacetate + 50 μg ethinyl estradiol	Developed SLE 4 weeks after the start of OCP and improved with withdrawal of OCP	(123)
Case report	22-year-old female	30 μg ethinyl estradiol + 250 μg levonorgestrel	Developed pulmonary hypertension related to SLE in 9 months	(121)
Case control study	109 SLE and 109 controls	OCP unspecified	No association between OCPs and SLE	(126)
Case report	24-year-old female	30 μg ethinyl estradiol	Developed malignant hypertension who has incomplete SLE with DNA antibodies and high levels of antiphospholipid antibodies	(119)
Case control study	195 SLE and 143 controls	OCP unspecified	No association between OCPs and SLE	(127)
Prospective cohort study	99 SLE confirmed among NHS cohort 121,645 women	Use of OCPs based on self-report	- Past users vs. never users: RR 1.9 (95% CI 1.1−3.3) - No relationship with duration of OCP use	(131)
Case control study	85 SLE and 205 controls	Use of OCPs containing estrogen based on self-report	No association between OCPs and SLE	(128)
Population-based case control study	240 SLE 240 and 321 controls	OCP unspecified	No association between OCPs and SLE	(129)
Prospective cohort study	262 SLE confirmed among NHS cohort 238,308 women	Use of OCPs based on self-report	- Ever use of OCPs: RR 1.5 (95% CI 1.1–2.1) - Highest risk with short duration (< 2 years) of OCPs: (RR 1.9, 95% CI 1.3–2.8)	(125)
Population based nested case control-study	786 SLE and 7,817 controls	COC exposure First- and second-generation (ethinyl estradiol combined with the progestatives norethisterone, levonorgestrel, and norgestrel) vs. third-generation (ethinyl estradiol and either gestodene, desogestrel, or norgestimate)	- Any use of OCPs: RR 1.19 (95% IC: 0.98–1.45) - Current use of OCPs: RR 1.54 (95% IC: 1.14–5.57) - Risk was higher in current users who recently started (RR 2.52, 95% CI: 1.14–5.57), first or second-generation OC (RR 1.65, 95% CI 1.20–2.26), and increase with dose of ethinyl estradiol (RR 1.42, 1.63, and 2.92 for ≤ 30 μg, 31−49 μg, and ≥ 50 μg, respectively)	(10)

SLE, systemic lupus erythematosus; COC, combined oral contraceptives; POC, progestin-only oral contraceptives; OCP, oral contraceptives; NHS, nurses’ health study; RR, relative risk; CI, confidence interval.

In addition to its association with disease activity, case reports and prospective cohort studies have reported newly diagnosed SLE after the initiation of OCPs (10, 119, 121–125). Sanchez-Guerrero et al. and Costenbader et al. reported associations between OCP use and SLE onset in 1997 and 2007, respectively, using the same population from the Nurses’ Health Study cohort (124, 125). The relative risk (RR) of OCP users compared to that of never users was 1.9 (95% confidence interval [CI] 1.1−3.3) in the first study and 1.4 (95% CI 0.9−2.1) in the second study. Another highly supportive study conducted in the United Kingdom evaluates the risk of SLE incidence related to COC use (10). In this study, COC use was associated with an increased risk of SLE (RR 1.19, 95% CI 0.98−1.45), especially in women who recently started using contraceptives or at higher doses. Malignant hypertension and vascular complication have also been reported in women with incomplete SLE featuring anti-DNA and antiphospholipid antibodies (119).

However, case-control studies have reported contradictory results (126–129). The first case-control study assessing the association between OCP exposure and risk of SLE was performed in 1985 with 109 cases of SLE and 109 HCs, reporting that recent use of OCPs was independent of SLE onset (odds ratio 0.5, 95% CI 0.11−2.3) (126). A case-control study from the United States with 195 cases of SLE and 143 HCs also showed no association between SLE and either any or recent use of OCPs (127). Although studies with similar results have been reported, limitations such as selection bias have hampered the precision of these studies (128, 129). Some studies have suggested that OCP use does not equally affect all individuals and triggers SLE by inducing antinuclear antibodies in predisposed individuals with autoimmune serologies (130).

Two higher-quality randomized controlled trials (RCT) were conducted to clarify this discrepancy between conflicting results (131, 132). The first RCT was a single-blinded, non-placebo study that followed 162 patients with stable SLE randomly assigned to a COC, intrauterine device, or progestin-only pill for 12 months. In this study, disease activity, flare incidence, and time-to-first flare did not differ significantly among the groups treated with different types of contraceptive therapy (131). The second double-blind RCT, the Safety of Estrogen in Lupus Erythematosus National Assessment (SELENA) study conducted in the United States, included 183 patients with inactive or stable active SLE randomly assigned to receive placebo or COC for 12 months (132). As with the previous RCT results, the flare rates were similar between the two groups, and the discontinuation rates did not differ for any reason.

Available evidence from RCTs supports the safety of OCPs, namely, COC, in most women with SLE. Based on these results, the World Health Organization published useful information regarding contraception for women with SLE, suggesting that most OCPs can be used by women with SLE if antiphospholipid antibodies are absent or cardiovascular risk factors are unclear (113). Since both RCTs excluded patients with SLE with unstable active diseases, the results may not be applicable to all women with SLE. Considering the data to date, the effects and risks of OCPs on SLE may vary depending on the dose, duration of use, and type of hormone used. Despite being theoretically possible, the use of OCPs should be fully discussed with respect to the balance of benefits and risks for each individual patient. Most importantly, OCPs are contraindicated for women with SLE displaying positive/unknown antiphospholipid antibodies or a history of thrombosis under all circumstances (133, 134).

#### Hormone replacement therapy and systemic lupus erythematosus

Hormone replacement therapy (HRT) is the most effective method for relieving menopausal symptoms such as vasomotor hot flashes, atrophic vaginitis, and urinary incontinence or frequency (135). In the 1980s and 1990s, early observational data supported that HRT reduced coronary heart disease and mortality, and initial data from the Women’s Health Initiative (WHI) RCT demonstrated a decreased incidence of osteoporosis-related fractures in postmenopausal women undergoing HRT (136, 137). Thus, HRT was widely used in menopausal women for 20 years. However, in the early 2000s, data from the WHI trial suggest that HRT was associated with an increased risk of invasive breast cancer, coronary heart disease, stroke, and pulmonary embolism, leading to an abrupt decline in the use of HRT in postmenopausal women worldwide (138, 139). Although the WHI was the largest RCT on HRT, only two hormone formulations were evaluated. Subsequent studies have changed the approach to HRT by evaluating different dosages and routes of estrogen therapy, namely, transdermal HRT or an ultra-low-dose oral product, and have recently demonstrated that the benefits outweigh the risks in women within 10 years of menopause and short-term use of low-dose HRT to alleviate menopause symptoms (140, 141).

Although HRT is generally considered to relieve menopausal symptoms in the short term and protect against chronic diseases in the long term for the general female population, many inconsistencies have been reported in patients with SLE. Table 2 lists the characteristics of studies investigating the relationship between HRT and SLE. A large prospective cohort study in 1995 reported that HRT was causally associated with an increased risk of developing SLE in postmenopausal women (142). In this study, the age-adjusted relative risks for the onset of SLE were 2.1 (95% CI 1.1−4.0) for ever users, 2.5 (CI 1.2−5.0) for current users, and 1.8 (CI, 0.8−4.1) for past users, compared with never users, and the duration of hormone use and risk of SLE were proportional. Additionally, a case-control study by Meier et al. reported that the risk of developing SLE increased as the duration of hormone use increased, and the magnitude of risk was related to estrogen dose (143). However, some studies have found no evidence of a significant increase in the incidence of SLE with HRT use (129).

**TABLE 2:** List of studies on the risk of disease onset or flares in patients with systemic lupus erythematosus using hormone replacement therapy.

Study design	Study population	Hormone replacement therapy, dose	Main findings	References
**SLE flares**				
Case control study	60 SLE (30 HRT users and age matched 30 never users)	HRT unspecified	- No differences between the two groups in ESR, hospital admission, or medications - HRT users experienced significant improvements in general wellbeing, libido and depression.	(144)
Case control study	48 SLE (16 HRT users and age matched 32 controls)	Estrogen dose (0.3−0.625 mg) and the progestogen dose (0−10 mg of MPA)	The use of HRT does not appear to increase the rate of flares (SLEDAI change) over a 1-year follow-up	(145)
Case control study	34 SLE (11 HRT and 23 non-HRT users)	0.625 mg of CEE (Days 1–21) and MPA 5 mg daily (Days 10–21)	No difference in flares (0.12 relapses/patient-year in HRT group vs. 0.16 relapses/patient-year in the non-HRT group, *p* = 0.90) and SLEDAI change (total SLEDAI score increase during flares/patient-year in the HRT and non-HRT groups were 0.55 and 1.22, respectively, *p* = 0.57) between two groups	(146)
Case report	64-year-old female	Estrogen for osteoporosis treatment	Flare of SLE in a 64-year-old woman in remission status after taking estrogen as a treatment for osteoporosis	(147)
Randomized, double-blind, placebo-controlled non-inferiority trial	351 menopausal patients with inactive (81.5%) or stable-active (18.5%) SLE	0.625 mg of CEE daily, plus MPA 5 mg for 12 days per month	- Mild to moderate flares were significantly increased in the HRT group: 1.14 flares/person-year for HRT and 0.86 flare/person-year for placebo (RR 1.34; *P* = 0.01) - HRT did not significantly increase the risk for severe flare compared with placebo	(9)
Double-blind, randomized clinical trial	106 SLE (52 HRT users and 54 placebo)	0.625 mg of conjugated estrogen daily, plus 5 mg of medroxyprogesterone for 10 days per month	- Menopause hormonal therapy did not alter disease activity (SLEDAI score) during 2 years of treatment - Increased risk of thrombosis in hormone therapy group	(148)
SLE onset				
Prospective cohort study	45 SLE confirmed among NHS cohort 69,435 women	Use of HRT based on self-report	- Ever uses of HRT: RR 2.1 (95% IC: 1.1–4.0) - Current uses of HRT: RR 2.5 (95% IC: 1.2–5.0) - Past use of HRT: RR 1.8 (95% IC: 0.8–4.1) - HRT is associated with an increased risk for developing SLE	(142)
Case control study	41 SLE, 34 discoid lupus, and 295 age- and sex-matched controls	HRT unspecified	- Developing SLE (adjusted OR 2.8; 95% CI 0.9–9.0) or discoid lupus (adjusted OR 2.8; 95% CI 1.0–8.3) who were exposed for 2 or more years - Increased risk in estrogen only (OR 5.3; 95% CI 1.5–18.6) rather than estrogen + progesterone (OR 2.0; 95% CI 0.8–5.0), compared to non-users.	(143)
Population-based case control study	240 SLE 240 and 321 controls	HRT unspecified	No association between HRT and SLE	(129)
Prospective cohort study	262 SLE confirmed among NHS cohort 238,308 women	Use of HRT based on self-report	Ever use of HRT: RR 1.9 (95% CI 1.2–3.1)	(125)

SLE, systemic lupus erythematosus; HRT, hormone replacement therapy; ESR, erythrocyte sedimentation rate; MPA, medroxyprogesterone acetate; SLEDAI, systemic lupus erythematosus disease activity index; CEE, conjugated equine estrogens; RR, relative risk; NHS, nurses’ for more details.

Several early retrospective case-control studies failed to find an association between flares and HRT (144–146). Although these were very small studies with insufficient data, the conclusion that HRT had a minimal effect on inflammatory markers and did not change disease activity (expressed by SLEDAI) was similar in all published observational studies. Conversely, case and prospective cohort studies have provided evidence linking HRT to flares (9, 147). In one case report, a woman diagnosed with SLE maintained remission status after menopause at 38 years of age but relapsed after taking estrogen as a treatment for osteoporosis at 64 years of age (147). The largest clinical trial to date investigating the effects of HRT on disease activity in patients with SLE was part of the SELENA trial, in which mild to moderate flares were significantly increased in the HRT group (9). However, neither the occurrence of severe flares nor the mean SLEDAI scores changed significantly between the HRT and placebo groups in this study. In another prospective study conducted by Sánchez-Guerrero et al. HRT use did not change the disease activity in SLE during 2 years of treatment (148).

As with taking OCPs, the greatest concern regarding HRT is the increased risk of arterial or venous thrombosis. Most studies on HRT in patients with SLE found a link between HRT use and thrombotic events (129, 144, 145, 148–151). Although the risk of developing thrombosis increases after HRT or menopause in healthy women, the incidence of thrombosis in women with SLE increased dramatically from 0.08 to 0.11 per 1,000 person/year to 5.1 per 1,000 person/year (149, 150, 152). Several RCTs reported that HRT use alone did not increase the risk of thrombosis or coronary heart disease for patients with SLE with inactive or stable active disease, negative antiphospholipid antibodies, and no history of thrombosis (9, 153, 154). The effects of hormones on thrombosis and the reported data indicate that HRT is not safe in patients with SLE with antiphospholipid antibodies or prior vascular thrombotic events. Smoking, old age, female sex, disease activity, and glucocorticoid dose are also known to increase the risk of thrombosis; therefore, HRT use should be cautioned for patients with these risk factors (155).

Furthermore, the influence of HRT on malignancy risk is a serious concern for women. In the general population, cancer risk was increased by 9% among users of HRT, which carried widely depending on the type of cancer and HRT regimen (156). In particular, the relationship between HRT and female reproductive organ cancers, such as breast, endometrial, and ovarian cancer, is of great interest. Fortunately, the risk of these cancers is rather decreased in women with SLE (157, 158). The tendency of patients with SLE to have a higher age at menarche and lower age at menopause compared to the general population leads to a decrease in lifetime estrogen exposure, reducing the incidence of female reproductive organ cancers (159). Studies on the causal relationship between cancer incidence and HRT in patients with SLE are rare, and the association between cancer and HRT in SLE has not yet been clarified in published studies (160).

In conclusion, HRT use needs to be individually tailored in consideration of various conditions. For women with SLE, transdermal or percutaneous estrogen formulations are preferred over oral preparations, and micronized progesterone or pregnane derivatives are preferred over non-pregnane when using combined estrogen and progesterone HRT. Moreover, if HRT is unavoidable in active disease, non-estrogenic drugs should be selected first (161).

## Conclusion

The effects of sex hormones, estrogen, and their receptors, especially ERα, have been found to promote autoimmune responses, namely, autoantibody production, and Th17 differentiation. In addition, DC activation and a type I IFN signature are modulated through TLR-7 and TLR-9 by estrogen or its receptor. While androgens inhibit B cell activation, testosterone and other androgens have not demonstrated therapeutic effects against SLE. Although sex hormones change during the menstrual cycle, flares rarely occur according to the menstrual cycle. Defective androgens are associated with male SLE, which is characterized by more frequent skin involvement and higher risk for renal damage. Although conflicting results have been reported regarding the use of OCPs and HRT in women with SLE, their use raises the risk of flares or cardiovascular diseases in patients with antiphospholipid antibodies or a history of thrombosis; therefore, hormone therapy for patients with SLE should be decided through close consultation.

## Data availability statement

The original contributions presented in this study are included in the article/supplementary material, further inquiries can be directed to the corresponding author/s.

## Author contributions

J-WK and J-YJ: conceptualization, methodology, formal analysis, investigation, data curation, writing—original draft preparation, review and editing, and project administration. J-WK: visualization. J-YJ: supervision and funding acquisition. All authors have read and agreed to the published version of the manuscript.

## Abbreviations

BAFF: B cell-activating factor
COC: combined oral contraceptives
DC: dendritic cell
dsDNA: double-stranded DNA
E2: estradiol
ER: estrogen receptor
FSH: follicle-stimulating hormone
HC: healthy control
HRT: hormone replacement therapy
OCP: oral contraceptive
PBMC: peripheral blood mononuclear cell
pDC: plasmacytoid DC
RCT: randomized controlled trial
SLE: systemic lupus erythematosus
SLEDAI: SLE disease activity index
WHI: Women’s Health Initiative.

## References

[1] Tsokos GC. Systemic lupus erythematosus. *N Engl J Med.* (2011) 365:2110–21. 10.1056/NEJMra1100359 22129255

[2] Kiriakidou M Ching CL. Systemic lupus erythematosus. *Ann Intern Med.* (2020) 172:Itc81–96. 10.7326/AITC202006020 32479157

[3] Christou EAA Banos A Kosmara D Bertsias GK Boumpas DT. Sexual dimorphism in SLE: above and beyond sex hormones. *Lupus.* (2019) 28:3–10. 10.1177/0961203318815768 30501463 PMC6304686

[4] Qin B Wang J Yang Z Yang M Ma N Huang F Epidemiology of primary Sjögren’s syndrome: a systematic review and meta-analysis. *Ann Rheum Dis.* (2015) 74:1983–9. 10.1136/annrheumdis-2014-205375 24938285

[5] Ortona E Pierdominici M Maselli A Veroni C Aloisi F Shoenfeld Y. Sex-based differences in autoimmune diseases. *Ann Ist Super Sanita.* (2016) 52:205–12. 27364395 10.4415/ANN_16_02_12

[6] Klein SL Flanagan KL. Sex differences in immune responses. *Nat Rev Immunol.* (2016) 16:626–38. 10.1038/nri.2016.90 27546235

[7] Petri M. Sex hormones and systemic lupus erythematosus. *Lupus.* (2008) 17:412–5. 10.1177/0961203308090026 18490418

[8] Kim JW Jung JY Kim HA Yang JI Kwak DW Suh CH. Lupus low disease activity state achievement is important for reducing adverse outcomes in pregnant patients with systemic lupus erythematosus. *J Rheumatol.* (2021) 48:707–16. 10.3899/jrheum.200802 33060317

[9] Buyon JP Petri MA Kim MY Kalunian KC Grossman J Hahn BH The effect of combined estrogen and progesterone hormone replacement therapy on disease activity in systemic lupus erythematosus: a randomized trial. *Ann Intern Med.* (2005) 142:953–62. 10.7326/0003-4819-142-12_Part_1-200506210-00004 15968009

[10] Bernier MO Mikaeloff Y Hudson M Suissa S. Combined oral contraceptive use and the risk of systemic lupus erythematosus. *Arthritis Rheum.* (2009) 61:476–81. 10.1002/art.24398 19333988

[11] Mok CC Lau CS Ho CT Wong RW. Do flares of systemic lupus erythematosus decline after menopause? *Scand J Rheumatol.* (1999) 28:357–62. 10.1080/03009749950155346 10665741

[12] Andrews BS Eisenberg RA Theofilopoulos AN Izui S Wilson CB McConahey PJ Spontaneous murine lupus-like syndromes. Clinical and immunopathological manifestations in several strains. *J Exp Med.* (1978) 148:1198–215. 10.1084/jem.148.5.1198 309911 PMC2185049

[13] Davey RA Grossmann M. Androgen receptor structure, function and biology: from bench to bedside. *Clin Biochem Rev.* (2016) 37:3–15. 27057074 PMC4810760

[14] Rothman MS Carlson NE Xu M Wang C Swerdloff R Lee P Reexamination of testosterone, dihydrotestosterone, estradiol and estrone levels across the menstrual cycle and in postmenopausal women measured by liquid chromatography-tandem mass spectrometry. *Steroids.* (2011) 76:177–82. 10.1016/j.steroids.2010.10.010 21070796 PMC3005029

[15] Hughes GC Choubey D. Modulation of autoimmune rheumatic diseases by oestrogen and progesterone. *Nat Rev Rheumatol.* (2014) 10:740–51. 10.1038/nrrheum.2014.144 25155581

[16] Cutolo M Straub RH. Sex steroids and autoimmune rheumatic diseases: state of the art. *Nat Rev Rheumatol.* (2020) 16:628–44. 10.1038/s41584-020-0503-4 33009519

[17] Billi AC Kahlenberg JM Gudjonsson JE. Sex bias in autoimmunity. *Curr Opin Rheumatol.* (2019) 31:53–61. 10.1097/BOR.0000000000000564 30394940 PMC6296774

[18] Elderman M de Vos P Faas M. Role of microbiota in sexually dimorphic immunity. *Front Immunol.* (2018) 9:1018. 10.3389/fimmu.2018.01018 PMC5992421 29910797

[19] Markle JG Frank DN Mortin-Toth S Robertson CE Feazel LM Rolle-Kampczyk U Sex differences in the gut microbiome drive hormone-dependent regulation of autoimmunity. *Science.* (2013) 339:1084–8. 10.1126/science.1233521 23328391

[20] Inui H Ogasawara T Naito Sekigawa I Takasaki Y Hayashida Y Takamori K Estrogen receptor expression by peripheral blood mononuclear cells of patients with systemic lupus erythematosus. *Clin Rheumatol.* (2007) 26:1675–8. 10.1007/s10067-007-0568-3 17874259

[21] Kassi EN Vlachoyiannopoulos PG Moutsopoulos HM Sekeris CE Moutsatsou P. Molecular analysis of estrogen receptor alpha and beta in lupus patients. *Eur J Clin Invest.* (2001) 31:86–93. 10.1046/j.1365-2362.2001.00762.x 11168443

[22] Bynoté KK Hackenberg JM Korach KS Lubahn DB Lane PH Gould KA. Estrogen receptor-alpha deficiency attenuates autoimmune disease in (NZB x NZW)F1 mice. *Genes Immun.* (2008) 9:137–52. 10.1038/sj.gene.6364458 18200028

[23] Svenson JL EuDaly J Ruiz P Korach KS Gilkeson GS. Impact of estrogen receptor deficiency on disease expression in the NZM2410 lupus prone mouse. *Clin Immunol.* (2008) 128:259–68. 10.1016/j.clim.2008.03.508 18514033 PMC4778964

[24] Grimaldi CM Cleary J Dagtas AS Moussai D Diamond B. Estrogen alters thresholds for B cell apoptosis and activation. *J Clin Invest.* (2002) 109:1625–33. 10.1172/JCI0214873 12070310 PMC151010

[25] Kanda N Tsuchida T Tamaki K. Estrogen enhancement of anti-double-stranded DNA antibody and immunoglobulin G production in peripheral blood mononuclear cells from patients with systemic lupus erythematosus. *Arthritis Rheum.* (1999) 42:328–37. 10.1002/1529-0131(199902)42:2<328::AID-ANR16>3.0.CO;2-# 10025928

[26] Grimaldi CM Jeganathan V Diamond B. Hormonal regulation of B cell development: 17 beta-estradiol impairs negative selection of high-affinity DNA-reactive B cells at more than one developmental checkpoint. *J Immunol.* (2006) 176:2703–10. 10.4049/jimmunol.176.5.2703 16493025

[27] Bynoe MS Grimaldi CM Diamond B. Estrogen up-regulates Bcl-2 and blocks tolerance induction of naive B cells. *Proc Natl Acad Sci USA.* (2000) 97:2703–8. 10.1073/pnas.040577497 10694576 PMC15993

[28] Sthoeger ZM Zinger H Mozes E. Beneficial effects of the anti-oestrogen tamoxifen on systemic lupus erythematosus of (NZBxNZW)F1 female mice are associated with specific reduction of IgG3 autoantibodies. *Ann Rheum Dis*. (2003) 62:341–6. 10.1136/ard.62.4.341 12634234 PMC1754513

[29] Panchanathan R Choubey D. Murine BAFF expression is upregulated by estrogen and interferons: implications for sex bias in the development of autoimmunity. *Mol Immunol.* (2013) 53:15–23. 10.1016/j.molimm.2012.06.013 22784990 PMC3439561

[30] Bassi N Luisetto R Ghirardello A Gatto M Valente M Della Barbera M 17-β-estradiol affects BLyS serum levels and the nephritogenic autoantibody network accelerating glomerulonephritis in NZB/WF1 mice. *Lupus.* (2015) 24:382–91. 10.1177/0961203314559636 25801881

[31] Rider V Li X Peterson G Dawson J Kimler BF Abdou NI. Differential expression of estrogen receptors in women with systemic lupus erythematosus. *J Rheumatol.* (2006) 33:1093–101. 16755656

[32] Rider V Jones SR Evans M Abdou NI. Molecular mechanisms involved in the estrogen-dependent regulation of calcineurin in systemic lupus erythematosus T cells. *Clin Immunol.* (2000) 95:124–34. 10.1006/clim.2000.4844 10779406

[33] Rider V Jones S Evans M Bassiri H Afsar Z Abdou NI. Estrogen increases CD40 ligand expression in T cells from women with systemic lupus erythematosus. *J Rheumatol.* (2001) 28:2644–9. 11764210

[34] Feng F Nyland J Banyai M Tatum A Silverstone AE Gavalchin J. The induction of the lupus phenotype by estrogen is *via* an estrogen receptor-alpha-dependent pathway. *Clin Immunol.* (2010) 134:226–36. 10.1016/j.clim.2009.10.004 19926524

[35] Kim DH Park HJ Park HS Lee JU Ko C Gye MC Estrogen receptor α in T cells suppresses follicular helper T cell responses and prevents autoimmunity. *Exp Mol Med.* (2019) 51:1–9. 10.1038/s12276-019-0237-z 30988419 PMC6465332

[36] Singh RP Bischoff DS. Sex hormones and gender influence the expression of markers of regulatory T Cells in SLE patients. *Front Immunol.* (2021) 12:619268. 10.3389/fimmu.2021.619268 PMC7966510 33746959

[37] Perry DJ Yin Y Telarico T Baker HV Dozmorov I Perl A Murine lupus susceptibility locus Sle1c2 mediates CD4+ T cell activation and maps to estrogen-related receptor gamma. *J Immunol.* (2012) 189:793–803. 10.4049/jimmunol.1200411 22711888 PMC3392454

[38] Li W Gong M Park YP Elshikha AS Choi SC Brown J Lupus susceptibility gene Esrrg modulates regulatory T cells through mitochondrial metabolism. *JCI Insight.* (2021) 6:e143540. 10.1172/jci.insight.143540 34156979 PMC8410062

[39] Lélu K Laffont S Delpy L Paulet PE Périnat T Tschanz SA Estrogen receptor α signaling in T lymphocytes is required for estradiol-mediated inhibition of Th1 and Th17 cell differentiation and protection against experimental autoimmune encephalomyelitis. *J Immunol.* (2011) 187:2386–93. 10.4049/jimmunol.1101578 21810607

[40] Chen RY Fan YM Zhang Q Liu S Li Q Ke GL Estradiol inhibits Th17 cell differentiation through inhibition of RORγT transcription by recruiting the ERα/REA complex to estrogen response elements of the RORγT promoter. *J Immunol.* (2015) 194:4019–28. 10.4049/jimmunol.1400806 25769926 PMC4390502

[41] Fuseini H Cephus JY Wu P Davis JB Contreras DC Gandhi VD ERα signaling increased IL-17A production in Th17 cells by upregulating IL-23R expression, mitochondrial respiration, and proliferation. *Front Immunol.* (2019) 10:2740. 10.3389/fimmu.2019.02740 PMC6892971 31849948

[42] Laffont S Seillet C Guéry JC. Estrogen receptor-dependent regulation of dendritic cell development and function. *Front Immunol.* (2017) 8:108. 10.3389/fimmu.2017.00108 PMC5300975 28239379

[43] Siracusa MC Overstreet MG Housseau F Scott AL Klein SL. 17beta-estradiol alters the activity of conventional and IFN-producing killer dendritic cells. *J Immunol.* (2008) 180:1423–31. 10.4049/jimmunol.180.3.1423 18209037

[44] Scott JL Cunningham MA Naga OS Wirth JR Eudaly JG Gilkeson GS. Estrogen receptor α deficiency modulates TLR ligand-mediated PDC-TREM expression in plasmacytoid dendritic cells in lupus-prone mice. *J Immunol.* (2015) 195:5561–71. 10.4049/jimmunol.1500315 26553076 PMC4790076

[45] Berghöfer B Frommer T Haley G Fink L Bein G Hackstein H. TLR7 ligands induce higher IFN-α production in females. *J Immunol.* (2006) 177:2088–96. 10.4049/jimmunol.177.4.2088 16887967

[46] Seillet C Laffont S Trémollières F Rouquié N Ribot C Arnal JF The TLR-mediated response of plasmacytoid dendritic cells is positively regulated by estradiol *in vivo* through cell-intrinsic estrogen receptor α signaling. *Blood.* (2012) 119:454–64. 10.1182/blood-2011-08-371831 22096248

[47] Cunningham MA Naga OS Eudaly JG Scott JL Gilkeson GS. Estrogen receptor α modulates toll-like receptor signaling in murine lupus. *Clin Immunol.* (2012) 144:1–12. 10.1016/j.clim.2012.04.001 22659029 PMC3737583

[48] Griesbeck M Ziegler S Laffont S Smith N Chauveau L Tomezsko P Sex differences in plasmacytoid dendritic cell levels of IRF5 drive higher IFN-α production in women. *J Immunol.* (2015) 195:5327–36. 10.4049/jimmunol.1501684 26519527 PMC4654231

[49] Young NA Wu LC Burd CJ Friedman AK Kaffenberger BH Rajaram MV Estrogen modulation of endosome-associated toll-like receptor 8: an IFNα-independent mechanism of sex-bias in systemic lupus erythematosus. *Clin Immunol* (2014) 151:66–77. 10.1016/j.clim.2014.01.006 24525049 PMC4066385

[50] Edwards MR Dai R Heid B Cowan C Werre SR Cecere T Low-dose 17alpha-ethinyl estradiol (EE) exposure exacerbates lupus renal disease and modulates immune responses to TLR7/9 agonists in genetically autoimmune-prone mice. *Sci Rep.* (2020) 10:5210. 10.1038/s41598-020-62124-6 32251357 PMC7090002

[51] Young NA Valiente GR Hampton JM Wu LC Burd CJ Willis WL Estrogen-regulated STAT1 activation promotes TLR8 expression to facilitate signaling *via* microRNA-21 in systemic lupus erythematosus. *Clin Immunol.* (2017) 176:12–22. 10.1016/j.clim.2016.12.005 28039018 PMC5815376

[52] Liu HW Lin HL Yen JH Tsai WC Chiou SS Chang JG Demethylation within the proximal promoter region of human estrogen receptor α gene correlates with its enhanced expression: implications for female bias in lupus. *Mol Immunol.* (2014) 61:28–37. 10.1016/j.molimm.2014.05.002 24861435

[53] Wu Z Sun Y Mei X Zhang C Pan W Shi W. 17β-oestradiol enhances global DNA hypomethylation in CD4-positive T cells from female patients with lupus, through overexpression of oestrogen receptor-α-mediated downregulation of DNMT1. *Clin Exp Dermatol.* (2014) 39:525–32. 10.1111/ced.12346 24825143

[54] Ramanujan SA Cravens EN Krishfield SM Kyttaris VC Moulton VR. Estrogen-induced hsa-miR-10b-5p is elevated in T cells from patients with systemic lupus erythematosus and down-regulates serine/arginine-rich splicing factor 1. *Arthritis Rheumatol.* (2021) 73:2052–8. 10.1002/art.41787 33982889 PMC8568617

[55] Dong G Fan H Yang Y Zhao G You M Wang T 17β-estradiol enhances the activation of IFN-α signaling in B cells by down-regulating the expression of let-7e-5p, miR-98-5p and miR-145a-5p that target IKKε. *Biochim Biophys Acta.* (2015) 1852:1585–98. 10.1016/j.bbadis.2015.04.019 25912736

[56] Papapavlou G Hellberg S Raffetseder J Brynhildsen J Gustafsson M Jenmalm MC Differential effects of estradiol and progesterone on human T cell activation *in vitro*. *Eur J Immunol.* (2021) 51:2430–40. 10.1002/eji.202049144 34223649

[57] Hughes GC Martin D Zhang K Hudkins KL Alpers CE Clark EA Decrease in glomerulonephritis and Th1-associated autoantibody production after progesterone treatment in NZB/NZW mice. *Arthritis Rheum.* (2009) 60:1775–84. 10.1002/art.24548 19479860

[58] Keisler LW Kier AB Walker SE. Effects of prolonged administration of the 19-nor-testosterone derivatives norethindrone and norgestrel to female NZB/W mice: comparison with medroxyprogesterone and ethinyl estradiol. *Autoimmunity.* (1991) 9:21–32. 10.3109/08916939108997120 1669844

[59] Wong AH Agrawal N Hughes GC. Altered IgG autoantibody levels and CD4(+) T cell subsets in lupus-prone Nba2 mice lacking the nuclear progesterone receptor. *Autoimmunity.* (2015) 48:389–401. 10.3109/08916934.2015.1030613 25857203 PMC4826613

[60] Wilhelmson AS Stubelius A Börjesson AE Wu J Stern A Malin S Androgens regulate bone marrow B lymphopoiesis in male mice by targeting osteoblast-lineage cells. *Endocrinology.* (2015) 156:1228–36. 10.1210/en.2014-1822 25643155

[61] Wilhelmson AS Lantero Rodriguez M Stubelius A Fogelstrand P Johansson I Buechler MB Testosterone is an endogenous regulator of BAFF and splenic B cell number. *Nat commun.* (2018) 9:2067. 10.1038/s41467-018-04408-0 29802242 PMC5970247

[62] Lahita RG Bradlow HL Ginzler E Pang S New M. Low plasma androgens in women with systemic lupus erythematosus. *Arthritis Rheum.* (1987) 30:241–8. 10.1002/art.1780300301 3032210

[63] Gordon C Wallace DJ Shinada S Kalunian KC Forbess L Braunstein GD Testosterone patches in the management of patients with mild/moderate systemic lupus erythematosus. *Rheumatology.* (2008) 47:334–8. 10.1093/rheumatology/kem342 18238794

[64] Lahita RG Cheng CY Monder C Bardin CW. Experience with 19-nortestosterone in the therapy of systemic lupus erythematosus: worsened disease after treatment with 19-nortestosterone in men and lack of improvement in women. *J Rheumatol.* (1992) 19:547–55. 1593576

[65] van Vollenhoven RF Park JL Genovese MC West JP McGuire JL. A double-blind, placebo-controlled, clinical trial of dehydroepiandrosterone in severe systemic lupus erythematosus. *Lupus.* (1999) 8:181–7. 10.1191/096120399678847588 10342710

[66] Petri MA Mease PJ Merrill JT Lahita RG Iannini MJ Yocum DE Effects of prasterone on disease activity and symptoms in women with active systemic lupus erythematosus. *Arthritis Rheum.* (2004) 50:2858–68. 10.1002/art.20427 15452837

[67] Ekblom-Kullberg S Kautiainen H Alha P Helve T Leirisalo-Repo M Julkunen H. Reproductive health in women with systemic lupus erythematosus compared to population controls. *Scand J Rheumatol.* (2009) 38:375–80. 10.1080/03009740902763099 19308803

[68] Ceccarelli F Orefice V Perrone G Pirone C Perricone C Truglia S Premature ovarian failure in patients affected by systemic lupus erythematosus: a cross-sectional study. *Clin Exp Rheumatol.* (2020) 38:450–4. 32083540

[69] Mayorga J Alpízar-Rodríguez D Prieto-Padilla J Romero-Díaz J Cravioto MC. Prevalence of premature ovarian failure in patients with systemic lupus erythematosus. *Lupus*. (2016) 25:675–83. 10.1177/0961203315622824 26678443

[70] Medeiros PB Febronio MV Bonfa E Borba EF Takiuti AD Silva CA. Menstrual and hormonal alterations in juvenile systemic lupus erythematosus. *Lupus.* (2009) 18:38–43. 10.1177/0961203308094652 19074167

[71] Colangelo K Haig S Bonner A Zelenietz C Pope J. Self-reported flaring varies during the menstrual cycle in systemic lupus erythematosus compared with rheumatoid arthritis and fibromyalgia. *Rheumatology.* (2011) 50:703–8. 10.1093/rheumatology/keq360 21115463

[72] Kawasaki M Sekigawa I Nozawa K Kaneko H Takasaki Y Takamori K Changes in the gene expression of peripheral blood mononuclear cells during the menstrual cycle of females is associated with a gender bias in the incidence of systemic lupus erythematosus. *Clin Exp Rheumatol.* (2009) 27:260–6. 19473566

[73] Brundin PMA Landgren BM Fjallstrom P Shamekh MM Gustafsson JA Johansson AF Expression of sex hormone receptor and immune response genes in peripheral blood mononuclear cells during the menstrual cycle. *Front Endocrinol.* (2021) 12:721813. 10.3389/fendo.2021.721813 PMC8493253 34630328

[74] Lu LJ Wallace DJ Ishimori ML Scofield RH Weisman MH. Review: Male systemic lupus erythematosus: a review of sex disparities in this disease. *Lupus.* (2010) 19:119–29. 10.1177/0961203309350755 19946032 PMC7304291

[75] Roubinian JR Talal N Greenspan JS Goodman JR Siiteri PK. Delayed androgen treatment prolongs survival in murine lupus. *J Clin Invest.* (1979) 63:902–11. 10.1172/JCI109390 447833 PMC372031

[76] Roubinian JR Talal N Greenspan JS Goodman JR Siiteri PK. Effect of castration and sex hormone treatment on survival, anti-nucleic acid antibodies, and glomerulonephritis in NZB/NZW F1 mice. *J Exp Med.* (1978) 147:1568–83. 10.1084/jem.147.6.1568 308087 PMC2184317

[77] Trigunaite A Khan A Der E Song A Varikuti S Jørgensen TN. Gr-1(high) CD11b+ cells suppress B cell differentiation and lupus-like disease in lupus-prone male mice. *Arthritis Rheum.* (2013) 65:2392–402. 10.1002/art.38048 23754362

[78] Jones JM Jorgensen TN. Androgen-mediated anti-inflammatory cellular processes as therapeutic targets in lupus. *Front Immunol.* (2020) 11:1271. 10.3389/fimmu.2020.01271 PMC7324484 32655565

[79] Stahl NI Decker JL. Androgenic status of males with systemic lupus erythematosus. *Arthritis Rheum.* (1978) 21:665–8. 10.1002/art.1780210609 736997

[80] Mok CC Lau CS. Profile of sex hormones in male patients with systemic lupus erythematosus. *Lupus.* (2000) 9:252–7. 10.1191/096120300680198926 10866095

[81] Mackworth-Young CG Parke AL Morley KD Fotherby K Hughes GR. Sex hormones in male patients with systemic lupus erythematosus: a comparison with other disease groups. *Eur J Rheumatol Inflamm.* (1983) 6:228–32. 6439564

[82] Olsen NJ Kovacs WJ. Case report: testosterone treatment of systemic lupus erythematosus in a patient with Klinefelter’s syndrome. *Am J Med Sci.* (1995) 310:158–60. 10.1097/00000441-199510000-00006 7573120

[83] Sasaki N Yamauchi K Sato R Masuda T Sawai T Inoue H. Klinefelter’s syndrome associated with systemic lupus erythematosus and autoimmune hepatitis. *Mod Rheumatol.* (2006) 16:305–8. 10.1007/s10165-006-0511-5 17039312

[84] Ocon A Peredo-Wende R Kremer JM Bhatt BD. Significant symptomatic improvement of subacute cutaneous lupus after testosterone therapy in a female-to-male transgender subject. *Lupus.* (2018) 27:347–8. 10.1177/0961203317734921 28992799

[85] Scofield RH Bruner GR Namjou B Kimberly RP Ramsey-Goldman R Petri M Klinefelter’s syndrome (47,XXY) in male systemic lupus erythematosus patients: support for the notion of a gene-dose effect from the X chromosome. *Arthritis Rheum.* (2008) 58:2511–7. 10.1002/art.23701 18668569 PMC2824898

[86] Liu K Kurien BT Zimmerman SL Kaufman KM Taft DH Kottyan LC X chromosome dose and sex bias in autoimmune diseases: increased prevalence of 47, XXX in systemic lupus erythematosus and Sjogren’s syndrome. *Arthritis Rheumatol.* (2016) 68:1290–300. 26713507 10.1002/art.39560 PMC5019501

[87] Dillon SP Kurien BT Li S Bruner GR Kaufman KM Harley JB Sex chromosome aneuploidies among men with systemic lupus erythematosus. *J Autoimmun.* (2012) 38:129–34. 10.1016/j.jaut.2011.10.004 PMC3309073 22154021

[88] Youness A Miqual C-H Guery J-C. Escape from X chromosome inactivation and the female predominance in autoimmune diseases. *Int J Mol Sci.* (2021) 22:1114. 10.3390/ijms22031114 PMC7865432 33498655

[89] Rees F Doherty M Grainge MJ Lanyon P Zhang W. The worldwide incidence and prevalence of systemic lupus erythematosus: a systematic review of epidemiological studies. *Rheumatology.* (2017) 56:1945–61. 10.1093/rheumatology/kex260 28968809

[90] Murphy G Isenberg D. Effect of gender on clinical presentation in systemic lupus erythematosus. *Rheumatology.* (2013) 52:2108–15. 10.1093/rheumatology/ket160 23641038

[91] Rees F Doherty M Grainge M Davenport G Lanyon P Zhang W. The incidence and prevalence of systemic lupus erythematosus in the UK, 1999-2012. *Ann Rheum Dis.* (2016) 75:136–41. 10.1136/annrheumdis-2014-206334 25265938 PMC4717400

[92] Garcia MA Marcos JC Marcos AI Pons-Estel BA Wojdyla D Arturi A Male systemic lupus erythematosus in a Latin-American inception cohort of 1214 patients. *Lupus.* (2005) 14:938–46. 10.1191/0961203305lu2245oa 16425573

[93] Soto ME Vallejo M Guillén F Simón JA Arena E Reyes PA. Gender impact in systemic lupus erythematosus. *Clin Exp Rheumatol.* (2004) 22:713–21. 15638045

[94] Sayhi S Achour TB Mezri S Hamdi MS Nour G Bilel A Clinical features of systemic lupus erythematosus in Tunisian males. *Curr Rheumatol Rev*. (2020) 16:139–42. 10.2174/1573397115666190916142945 32423372

[95] Renau AI Isenberg DA. Male versus female lupus: a comparison of ethnicity, clinical features, serology and outcome over a 30 year period. *Lupus.* (2012) 21:1041–8. 10.1177/0961203312444771 22505605

[96] Riveros Frutos A Casas I Rúa-Figueroa I López-Longo FJ Calvo-Alén J Galindo M Systemic lupus erythematosus in Spanish males: a study of the Spanish Rheumatology Society Lupus Registry (RELESSER) cohort. *Lupus.* (2017) 26:698–706. 10.1177/0961203316673728 27799439

[97] Andrade RM Alarcón GS Fernández M Apte M Vilá LM Reveille JD. Accelerated damage accrual among men with systemic lupus erythematosus: XLIV. Results from a multiethnic US cohort. *Arthritis Rheum.* (2007) 56:622–30. 10.1002/art.22375 17265497

[98] Tabor DE Gould KA. Estrogen receptor alpha promotes lupus in (NZB×NZW)F1 mice in a B cell intrinsic manner. *Clin Immunol.* (2017) 174:41–52. 10.1016/j.clim.2016.10.011 27989899 PMC5316311

[99] Tan TC Fang H Magder LS Petri MA. Differences between male and female systemic lupus erythematosus in a multiethnic population. *J Rheumatol.* (2012) 39:759–69. 10.3899/jrheum.111061 22382348 PMC3605704

[100] Shaharir SS Kadir WDA Nordin F Bakar FA Ting MWH Jamil A Systemic lupus erythematosus among male patients in Malaysia: how are we different from other geographical regions? *Lupus.* (2019) 28:137–44. 10.1177/0961203318812676 30458692

[101] Voulgari PV Katsimbri P Alamanos Y Drosos AA. Gender and age differences in systemic lupus erythematosus. A study of 489 Greek patients with a review of the literature. *Lupus.* (2002) 11:722–9. 10.1191/0961203302lu253oa 12475002

[102] Stefanidou S Benos A Galanopoulou V Chatziyannis I Kanakoudi F Aslanidis S Clinical expression and morbidity of systemic lupus erythematosus during a post-diagnostic 5-year follow-up: a male:female comparison. *Lupus.* (2011) 20:1090–4. 10.1177/0961203311403640 21700658

[103] de Carvalho JF do Nascimento AP Testagrossa LA Barros RT Bonfá E. Male gender results in more severe lupus nephritis. *Rheumatol Int.* (2010) 30:1311–5. 10.1007/s00296-009-1151-9 19784840

[104] Boodhoo KD Liu S Zuo X. Impact of sex disparities on the clinical manifestations in patients with systemic lupus erythematosus: A systematic review and meta-analysis. *Medicine.* (2016) 95:e4272. 10.1097/MD.0000000000004272 27442661 PMC5265778

[105] Feldman CH Broder A Guan H Yazdany J Costenbader KH. Sex differences in health care utilization, end-stage renal disease, and mortality among Medicaid Beneficiaries with incident lupus nephritis. *Arthritis Rheumatol.* (2018) 70:417–26. 10.1002/art.40392 29193893 PMC5826885

[106] Aranow C Del Guidice J Barland P Weinstein A. Systemic lupus erythematosus disease severity in men and women: a case-control study. *J Rheumatol*. (2002) 29:1674–7. 12180728

[107] Hwang J Lee J Ahn JK Park EJ Cha HS Koh EM. Clinical characteristics of male and female Korean patients with systemic lupus erythematosus: a comparative study. *Korean J Intern Med.* (2015) 30:242–9. 10.3904/kjim.2015.30.2.242 25750567 PMC4351332

[108] Hsu CY Chiu WC Yang TS Chen CJ Chen YC Lai HM Age- and gender-related long-term renal outcome in patients with lupus nephritis. *Lupus.* (2011) 20:1135–41. 10.1177/0961203311404912 21719527

[109] Peng W Tang Y Tan L Qin W. Clinicopathological study of male and female patients with lupus nephritis: a retrospective study. *Int Urol Nephrol.* (2018) 50:313–20. 10.1007/s11255-017-1780-y 29299823

[110] Lin WH Guo CY Wang WM Yang DC Kuo TH Liu MF Incidence of progression from newly diagnosed systemic lupus erythematosus to end stage renal disease and all-cause mortality: a nationwide cohort study in Taiwan. *Int J Rheum Dis.* (2013) 16:747–53. 10.1111/1756-185X.12208 24382283

[111] Birru Talabi M Himes KP Clowse MEB. Optimizing reproductive health management in lupus and Sjogren’s syndrome. *Curr Opin Rheumatol.* (2021) 33:570–8. 10.1097/BOR.0000000000000839 34519280

[112] Christin-Maitre S. History of oral contraceptive drugs and their use worldwide. *Best Pract Res Clin Endocrinol Metab*. (2013) 27:3–12. 10.1016/j.beem.2012.11.004 23384741

[113] Benagiano G Benagiano M Bianchi P D’Elios MM Brosens I. Contraception in autoimmune diseases. *Best Pract Res Clin Obstet Gynaecol.* (2019) 60:111–23. 10.1016/j.bpobgyn.2019.05.003 31160225

[114] Petri M. Exogenous estrogen in systemic lupus erythematosus: oral contraceptives and hormone replacement therapy. *Lupus.* (2001) 10:222–6. 10.1191/096120301676707393 11315357

[115] Pimstone B. Systemic lupus erythematosus exacerbated by oral contraceptives. *S Afr Med J.* (1966) 4:62–3.

[116] Chapel TA Burns R. Oral contraceptives and exacerbation of lupus erythematosus. *Am J Obstet Gynecol*. (1971) 110:366–9. 10.1016/0002-9378(71)90730-7 5088375

[117] Jungers P Dougados M Pélissier C Kuttenn F Tron F Lesavre P Influence of oral contraceptive therapy on the activity of systemic lupus erythematosus. *Arthritis Rheum.* (1982) 25:618–23. 10.1002/art.1780250603 7092961

[118] Miller MH. Pulmonary hypertension, systemic lupus erythematosus, and the contraceptive pill: another report. *Ann Rheum Dis.* (1987) 46:159–61. 10.1136/ard.46.2.159 3827339 PMC1002084

[119] Julkunen H Kaaja R Jouhikainen T Teppo AM Friman C. Malignant hypertension and antiphospholipid antibodies as presenting features of SLE in a young woman using oral contraceptives. *Br J Rheumatol.* (1991) 30:471–2. 10.1093/rheumatology/30.6.471 1747706

[120] Buyon JP Kalunian KC Skovron ML Petri M Lahita R Merrill J Can women with systemic lupus erythematosus safely use exogenous estrogens? *J Clin Rheumatol.* (1995) 1:205–12. 10.1097/00124743-199508000-00002 19077980

[121] Todd GR McAteer EJ Jack CM Haire M Roberts SD Buchanan KD. Pulmonary hypertension, systemic lupus erythematosus, and the contraceptive pill. *Ann Rheum Dis.* (1985) 44:266–7. 10.1136/ard.44.4.266 3985693 PMC1001624

[122] Travers RL Hughes GR. Oral contraceptive therapy and systemic lupus erythematosus. *J Rheumatol.* (1978) 5:448–51. 310887

[123] Garovich M Agudelo C Pisko E. Oral contraceptives and systemic lupus erythematosus. *Arthritis Rheum.* (1980) 23:1396–8. 10.1002/art.1780231213 7458971

[124] Sanchez-Guerrero J Karlson EW Liang MH Hunter DJ Speizer FE Colditz GA. Past use of oral contraceptives and the risk of developing systemic lupus erythematosus. *Arthritis Rheum.* (1997) 40:804–8. 10.1002/art.1780400505 9153539

[125] Costenbader KH Feskanich D Stampfer MJ Karlson EW. Reproductive and menopausal factors and risk of systemic lupus erythematosus in women. *Arthritis Rheum.* (2007) 56:1251–62. 10.1002/art.22510 17393454

[126] Grimes DA LeBolt SA Grimes KR Wingo PA. Systemic lupus erythematosus and reproductive function: a case-control study. *Am J Obstet Gynecol.* (1985) 153:179–86. 10.1016/0002-9378(85)90108-5 4037012

[127] Strom BL Reidenberg MM West S Snyder ES Freundlich B Stolley PD. Shingles, allergies, family medical history, oral contraceptives, and other potential risk factors for systemic lupus erythematosus. *Am J Epidemiol.* (1994) 140:632–42. 10.1093/oxfordjournals.aje.a117302 7942763

[128] Bengtsson AA Rylander L Hagmar L Nived O Sturfelt G. Risk factors for developing systemic lupus erythematosus: a case-control study in southern Sweden. *Rheumatology.* (2002) 41:563–71. 10.1093/rheumatology/41.5.563 12011382

[129] Cooper GS Dooley MA Treadwell EL St Clair EW Gilkeson GS. Hormonal and reproductive risk factors for development of systemic lupus erythematosus: results of a population-based, case-control study. *Arthritis Rheum.* (2002) 46:1830–9. 10.1002/art.10365 12124867

[130] Kennedy JM. Oral contraceptives and ANA positivity. *Arthritis Rheum.* (1977) 20:1567–9. 10.1002/art.1780200832 303521

[131] Sánchez-Guerrero JS Uribe AG Jiménez-Santana L Mestanza-Peralta M Lara-Reyes P Seuc AH A trial of contraceptive methods in women with systemic lupus erythematosus. *N Engl J Med.* (2005) 353:2539–49. 10.1056/NEJMoa050817 16354890

[132] Petri M Kim MY Kalunian KC Grossman J Hahn BH Sammaritano LR Combined oral contraceptives in women with systemic lupus erythematosus. *N Engl J Med.* (2005) 353:2550–8. 10.1056/NEJMoa051135 16354891

[133] Duarte C Inês L. Oral contraceptives and systemic lupus erythematosus: what should we advise to our patients? *Acta Reumatol Port.* (2010) 35:133–40. 20711088

[134] Rojas-Villarraga A Torres-Gonzalez JV Ruiz-Sternberg M. Safety of hormonal replacement therapy and oral contraceptives in systemic lupus erythematosus: a systematic review and meta-analysis. *PLoS One.* (2014) 9:e104303. 10.1371/journal.pone.0104303 PMC4138076 25137236

[135] Lobo RA. Hormone-replacement therapy: current thinking. *Nat Rev Endocrinol.* (2017) 13:220–31. 10.1038/nrendo.2016.164 27716751

[136] Wolf PH Madans JH Finucane FF Higgins M Kleinman JC. Reduction of cardiovascular disease—related mortality among postmenopausal women who use hormones: evidence from a national cohort. *Am J Obstet Gynecol.* (1991) 164:489–94. 10.1016/S0002-9378(11)80006-2 1992690

[137] Cauley JA Robbins J Chen Z Cummings SR Jackson RD LaCroix AZ Effects of estrogen plus progestin on risk of fracture and bone mineral density: the Women’s Health Initiative randomized trial. *JAMA.* (2003) 290:1729–38. 10.1001/jama.290.13.1729 14519707

[138] Rossouw JE Anderson GL Prentice RL LaCroix AZ Kooperberg C Stefanick ML Writing Group for the Women’s Health Initiative Investigators. Risks and benefits of estrogen plus progestin in healthy postmenopausal women: principal results from the Women’s Health Initiative randomized controlled trial. *JAMA.* (2002) 288:321–33. 10.1001/jama.288.3.321 12117397

[139] Anderson GL Limacher M Assaf AR Bassford T Beresford SA Black H Effects of conjugated equine estrogen in postmenopausal women with hysterectomy: the Women’s Health Initiative randomized controlled trial. *JAMA.* (2004) 291:1701–12. 10.1001/jama.291.14.1701 15082697

[140] Lundberg G Wu P Wenger N. Menopausal hormone therapy: a comprehensive review. *Curr Atheroscler Rep.* (2020) 22:33. 10.1007/s11883-020-00854-8 32556827

[141] Palacios S Stevenson JC Schaudig K Lukasiewicz M Graziottin A. Hormone therapy for first-line management of menopausal symptoms: practical recommendations. *Womens Health.* (2019) 15:1745506519864009. 10.1177/1745506519864009 PMC6683316 31378196

[142] Sánchez-Guerrero JS Liang MH Karlson EW Hunter DJ Colditz GA. Postmenopausal estrogen therapy and the risk for developing systemic lupus erythematosus. *Ann Intern Med.* (1995) 122:430–3. 10.7326/0003-4819-122-6-199503150-00005 7856991

[143] Meier CR Sturkenboom MC Cohen AS Jick H. Postmenopausal estrogen replacement therapy and the risk of developing systemic lupus erythematosus or discoid lupus. *J Rheumatol.* (1998) 25:1515–9. 9712093

[144] Arden N Lloyd M Spector T Hughes G. Safety of hormone replacement therapy (HRT) in systemic lupus erythematosus (SLE). *Lupus.* (1994) 3:11–3. 10.1177/096120339400300104 8025579

[145] Kreidstein S Urowitz M Gladman D Gough J. Hormone replacement therapy in systemic lupus erythematosus. *J Rheumatol.* (1997) 24:2149–52. 9375875

[146] Mok CL Ho CTK Lee KW Mok MY Wong RWS Safety CC. of hormonal replacement therapy in postmenopausal patients with systemic lupus erythematosus. *Scand J Rheumatol.* (1998) 27:342–6. 10.1080/03009749850154357 9808396

[147] Barrett C Neylon N Snaith M. Oestrogen-induced systemic lupus erythematosus. *Rheumatology.* (1986) 25:300–1. 10.1093/rheumatology/25.3.300 3730740

[148] Sánchez-Guerrero JS González-Pérez M Durand-Carbajal M Lara-Reyes P Jiménez-Santana L Romero-Díaz J Menopause hormonal therapy in women with systemic lupus erythematosus. *Arthritis Rheum.* (2007) 56:3070–9. 10.1002/art.22855 17763408

[149] Somers E Magder LS Petri M. Antiphospholipid antibodies and incidence of venous thrombosis in a cohort of patients with systemic lupus erythematosus. *J Rheumatol.* (2002) 29:2531–6. 12465147

[150] Sarabi ZS Chang E Bobba R Ibanez D Gladman D Urowitz M Incidence rates of arterial and venous thrombosis after diagnosis of systemic lupus erythematosus. *Arthritis Rheum.* (2005) 53:609–12. 10.1002/art.21314 16082635

[151] Hulley S Grady D Bush T Furberg C Herrington D Riggs B Randomized trial of estrogen plus progestin for secondary prevention of coronary heart disease in postmenopausal women. Heart and Estrogen/progestin Replacement Study (HERS) Research Group. *JAMA.* (1998) 280:605–13. 10.1001/jama.280.7.605 9718051

[152] Daly E Vessey MP Hawkins MM Carson JL Gough P Marsh S. Risk of venous thromboembolism in users of hormone replacement therapy. *Lancet.* (1996) 348:977–80. 10.1016/S0140-6736(96)07113-9 8855852

[153] Hochman J Urowitz MB Ibañez D Gladman DD. Hormone replacement therapy in women with systemic lupus erythematosus and risk of cardiovascular disease. *Lupus.* (2009) 18:313–7. 10.1177/0961203308097475 19276299

[154] Fernández M Calvo-Alén J Bertoli AM Bastian HM Fessler BJ McGwin G Jr. Systemic lupus erythematosus in a multiethnic US cohort (LUMINA L II): relationship between vascular events and the use of hormone replacement therapy in postmenopausal women. *J Clin Rheumatol.* (2007) 13:261–5. 10.1097/RHU.0b013e318156bbf5 17921793

[155] Grygiel-Górniak B Puszczewicz MJ. The influence of endogenous and exogenous sex hormones on systemic lupus erythematosus in pre- and postmenopausal women. *Prz Menopauzalny.* (2014) 13:262–6. 10.5114/pm.2014.45003 26327864 PMC4520373

[156] Simin J Tamimi R Lagergren J Adami HO Brusselaers N. Menopausal hormone therapy and cancer risk: an overestimated risk? *Eur J Cancer.* (2017) 84:60–8. 10.1016/j.ejca.2017.07.012 28783542

[157] D’Alonzo M Bounous VE Villa M Biglia N. Current evidence of the oncological benefit-risk profile of hormone replacement therapy. *Medicina.* (2019) 55:573. 10.3390/medicina55090573 31500261 PMC6780494

[158] Fanouriakis A Tziolos N Bertsias G Boumpas DT. Update *o*n the diagnosis and management of systemic lupus erythematosus. *Ann Rheum Dis.* (2021) 80:14–25. 10.1136/annrheumdis-2020-218272 33051219

[159] Ladouceur A Clarke AE Ramsey-Goldman R Bernatsky S. Malignancies in systemic lupus erythematosus: an update. *Curr Opin Rheumatol.* (2019) 31:678–81. 10.1097/BOR.0000000000000648 31403485

[160] Bernatsky S Clarke A Ramsey-Goldman R Joseph L Boivin JF Rajan R Hormonal exposures and breast cancer in a sample of women with systemic lupus erythematosus. *Rheumatology.* (2004) 43:1178–81. 10.1093/rheumatology/keh282 15226516

[161] Gompel A Piette JC. Systemic lupus erythematosus and hormone replacement therapy. *Menopause Int.* (2007) 13:65–70. 10.1258/175404507780796433 17540136

